# Clinical Profiles and Outcomes of Heart Failure in Five African Countries: Results from INTER-CHF Study

**DOI:** 10.5334/gh.940

**Published:** 2021-07-30

**Authors:** Kamilu M. Karaye, Hisham Dokainish, Ahmed ElSayed, Charles Mondo, Albertino Damasceno, Karen Sliwa, Kumar Balasubramanian, Alex Grinvalds, Salim Yusuf, on behalf of investigators of INTERnational Congestive Heart Failure (INTER-CHF) Study

**Affiliations:** 1 Department of Medicine, Bayero University/Aminu Kano Teaching Hospital, Kano, NG; 2 Department of Public Health and Clinical Medicine, Umea University, SE; 3 Population Health Research Institute, McMaster University, Hamilton, CA; 4 Alazhari Health Research Center, Alzaeim Alazhari University/AlShaab Teaching Hospital, Khartoum, SD; 5 Mulago National Referral Hospital, Kampala, UG; 6 Faculty of Medicine, Eduardo Mondlane University, Maputo, MZ; 7 Hatter Institute for Cardiovascular Research in Africa & Cape Heart Institute, Faculty of Health Sciences, University of Cape Town, ZA

**Keywords:** Heart Failure, Africa, Etiology, Mortality, Hospitalization, INTER-CHF

## Abstract

**Background:**

A wide knowledge gap exists on the clinical profiles and outcomes of heart failure (HF) in sub-Saharan Africa.

**Objectives:**

To determine the clinical profiles and outcomes of HF patients from five African countries.

**Methods:**

The INTERnational Congestive Heart Failure Study (INTER-CHF) is a prospective, multicenter cohort study. A total of 1,294 HF patients were consecutively recruited from Nigeria (383 patients), South Africa (169 patients), Sudan (501 patients), Uganda (151patients), and Mozambique (90 patients). HF was defined according to the Boston criteria for diagnosis. Cognitive function was assessed using the Montreal Cognitive Assessment (MoCA) score.

**Results:**

Of the 1294 patients, 51.4% were recruited as out-patients, 53.7% had HF with reduced ejection fraction (EF), 30.1% had HF with mid-range EF and 16.2% had HF with preserved EF (16.2%). The commonest etiologies of HF were hypertensive heart disease (35%) and ischemic heart disease (20%). The mean MoCA score was highest in Uganda (24.3 ± 1.1) and lowest in Sudan (13.6 ± 0.3). Prescriptions for guideline-recommended HF therapies were poor; only 1.2% of South African patients received an Implantable Cardioverter Defibrillator, and none of the patients received Cardiac Resynchronised Therapy. The composite outcome of death or HF hospitalization at one year among the patients was highest in Sudan (59.7%) and lowest in Mozambique (21.1%). Six variables were associated with higher mortality risk, while digoxin use (adjusted hazard ratio [aHR]: 0.69; 95% confidence interval [CI]: 0.49–0.97; p = 0.034) and 10mmHg unit increase in systolic blood pressure (aHR 0.86; 95%CI 0.81–0.93; p < 0.001) were associated with lower risk for mortality.

**Conclusions:**

This is the largest HF study in Africa that included in- and out-patients from the West, East, North, Central and South African sub-regions. Clinically relevant differences, including cognitive functional impairment, were found between the involved countries.

## Introduction

Heart failure (HF) is a major health problem affecting about 64.3 million persons globally, with an epidemiology that varies widely within and between countries [1]. The INTERnational Congestive Heart Failure Study (INTER-CHF) was an international, multicenter cohort study, conducted in sixteen countries in Africa, Asia, the Middle East, and South America with six- and twelve-month follow-ups [2,3,4]. It was the first major study to systematically acquire data on in- and out-patients with HF in these regions, which were hitherto under-represented in previous global HF studies. It revealed that mortality rate was highest in Africa (34%), intermediate in southeast Asia (15%), and lowest in China (7%), South America (9%), and the Middle East (9%), and the regional differences persisted after multivariable adjustment [4]. The investigators believed that the variations in mortality between the regions were due to an interplay between health-care infrastructure, and environmental and genetic factors [4].

Prior to the INTER-CHF study, investigators of The Sub-Saharan Africa Survey of Heart Failure (THESUS–HF) had commendably reported on the morbidity and mortality of 1006 acute HF patients from ten African countries, but 42.7% of the patients were recruited from Nigeria alone and the follow-up period was relatively short (180 days) [5]. In this study, the median in-hospital and 180-day mortality rates were 4.2% and 17.8% respectively. Thus, larger and longer term studies from Africa involving patients with more varied HF phenotypes are desirable.

In this paper, we present results from the INTER-CHF study on the clinical profiles and outcomes of a sample of HF patients from Nigeria, Sudan, Mozambique, Uganda and South Africa; representing the five African regions. We assessed all-cause deaths, HF hospitalizations and the composite of the deaths or HF hospitalizations, over one year. We hypothesize that there could be differences between the five African countries in terms of the HF patients’ characteristics, etiology, treatment patterns and outcomes given the regional disparities seen in the main INTER-CHF study.

## Methods

**Figure 1:**
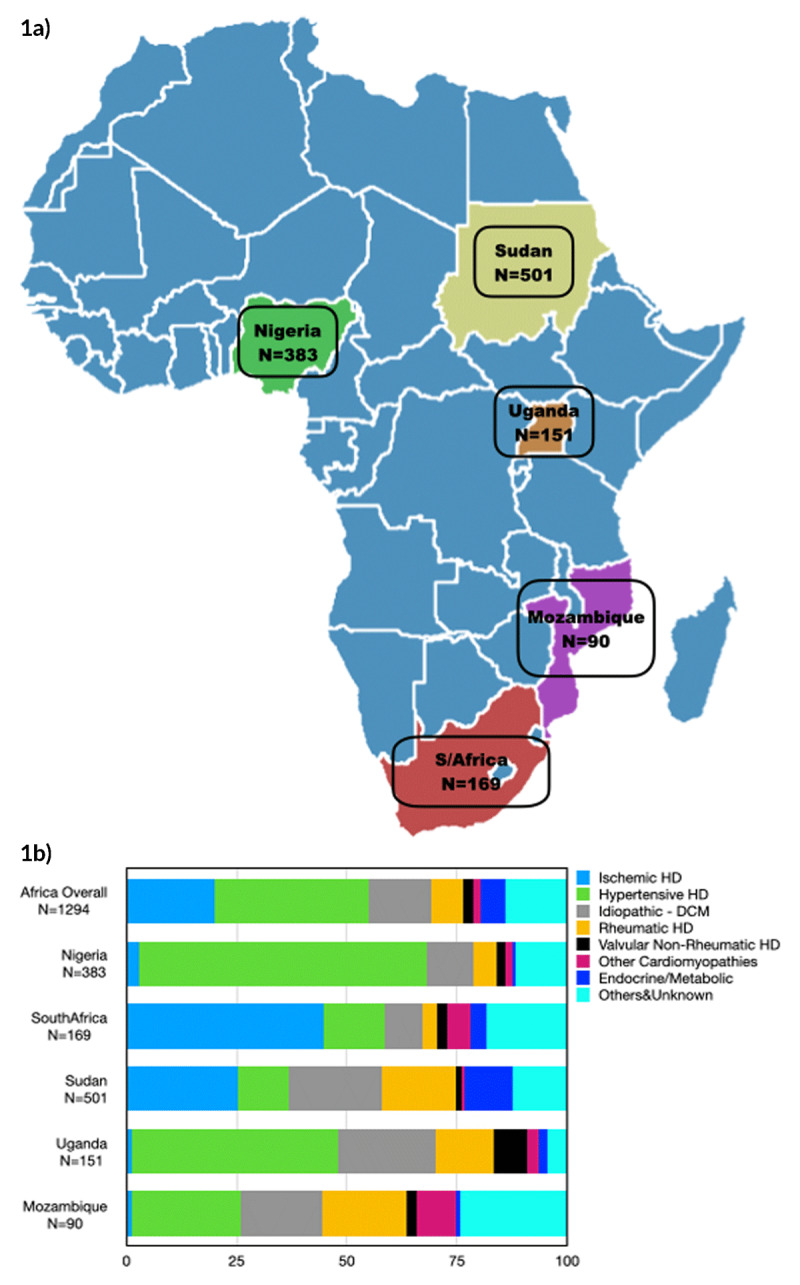
**(a)** INTER-CHF study sites. **(b)** Common etiologies of Heart Failure in the 5 African countries. **a)** Legend: African countries involved in INTER-CHF with recruitment numbers: Nigeria (383 patients), South Africa (169 patients), Sudan (501 patients), Uganda (151 patients), and Mozambique (90 patients). Total recruitment (1,294 patients). **b)** Legend: Prevalence of common etiologies of heart failure in the five African countries in INTER-CHJF study. It shows that hypertensive heart disease (HD) was the most common etiology in Nigeria, Uganda, Mozambique and overall, while ischemic HD was the most common in South Africa and Sudan.

INTER-CHF was a prospective, multicenter cohort study, conducted in 108 centers in 16 countries in Africa, Asia, the Middle East, and South America with 12 months follow-up [2]. A total of 1,294 HF patients were recruited from the five African regions: Nigeria (383 patients) (West Africa), South Africa (169 patients), Sudan (501 patients) (North Africa), Uganda (151 patients) (Central Africa), and Mozambique (90 patients) (East Africa) (Figure 1a). The rationale and design, baseline characteristics and main outcome results of the INTER-CHF study have already been published [2,3,4].

HF was defined according to the Boston criteria for diagnosis, which had been validated as a diagnostic tool for HF [6,7]. The inclusion criteria were age ≥18 years old and written informed consent from all study participants. The exclusion criteria were patients considered unreliable by the investigator for follow-up visits, and patients with a life expectancy less than the expected duration of the registry due to non-HF co-morbidities [2].

We aimed to recruit two-thirds of the HF patients from out-patient clinics and the remaining one-third from in-patient hospital wards at participating centres. Patients were consecutively identified and were aged 18 years or older and enrolled from academic/specialized health-care centres, community health centres, and specialist and primary care clinics. We excluded patients with severe non-cardiac diseases that could affect survival within one year, and patients who were difficult to follow-up (for example, because of lack of reliable contact telephone numbers or migratory status).

The study was approved by institutional review boards or independent ethics committees at participating sites and the international coordinating centre (Population Health Research Institute, McMaster University, Hamilton, Canada). Participants or their substitute decision maker provided written informed consent.

### Procedures

At enrolment, we recorded information on demographic and clinical features, medications, and socioeconomic factors. HF was classified using LV ejection fraction (LVEF) on echocardiogram as ‘reduced’ (HFrEF) when less than 40%, ‘mid-range’ (HFmrEF) when between 40 to 49%, and ‘preserved’ (HFpEF) when 50% or higher, in accordance with the recommendation of European Society of Cardiology (ESC) guidelines [8]. In INTER-CHF study, determination of HF etiology was at the discretion of the recruiting physician, based on the recommendations of standard practice guidelines, and using all available clinical, laboratory and echocardiographic data [3]. Valvular heart disease was defined as the presence of at least moderate stenosis or regurgitation in at least one cardiac valve. Cognitive function was assessed using the Montreal Cognitive Assessment (MoCA) score. We assessed the different cognitive domains: attention and concentration, executive functions, memory, language, visuo-constructional skills, conceptual thinking, calculations and orientation. The total possible score is 30 points and a score of 26 or above is considered normal [2].

Local physicians determined the causes of HF using all available clinical data and standard international practice guidelines. Patients had follow-up visits at 6 months and 12 months, at which time symptoms, medications and outcomes were recorded.

### Outcomes

Primary outcome was time to all-cause mortality within one year. Cause of death was also recorded, and categorised by local investigators as cardiac, other causes, or unknown. We also assessed HF hospitalizations and the composite of the deaths or HF hospitalizations, over one year.

### Statistical analysis

Baseline characteristics were described using summary statistics adjusted for age and sex. Inter-region comparisons for baseline characteristics were compared using ANOVA for continuous variables or chi-squared tests for categorical variables. Time to event analysis was used for all-cause mortality using the Kaplan Meier method followed by a multivariable Cox-Proportional Hazard Model (proportional hazards confirmed by visual inspection) to derive adjusted hazard ratios (aHR) and 95% Confidence Interval; with consideration of baseline demographic and clinical profile during follow-up as independent determinants of all-cause mortality. Two-sided p-value < 0.05 were considered statistically significant. The statistical analysis was performed using SAS version 9.4 for UNIX (SAS Institute Inc, Cary, NC, USA).

## Results

In the INTER-CHF study, a total of 1,294 HF patients were recruited from the five African countries between September 2012 and February 2014, representing 22.2% of the global cohort (Figure 1a), and followed up for a median of 368 (25^th^ and 75^th^ quartiles: 198–395) days. In the African cohort, 51.9% were males, 48.6% were recruited during a HF hospitalization, 42.9% were illiterate, 66.9% had no health insurance, 29.6% were recruited from Nigeria, 13.1% from South Africa, 38.7% from Sudan, 11.7% from Uganda, and 7.0% from Mozambique. Details of the baseline characteristics and HF medications are presented in Table 1 (and Supplementary file) and compared between the five countries. It shows significant differences in the profiles of HF patients in the five countries.

**Table 1:** Baseline characteristics and treatments.

Variables	Overall Mean(SE)or n(%) N = 5823	Africa Overall Mean(SE)or n(%) N = 1294	Nigeria Mean(SE)or n(%) N = 383	SouthAfrica Mean(SE)or(%) N = 169	Sudan Mean(SE)or n(%) N = 501	Uganda Mean(SE)or n(%) N = 151	Mozambique Mean(SE) or n(%) N = 90

**Demographic characteristics**							

Age, years	59.3(0.2)	53.4(0.4)	50.8(0.8)	53.3(1.2)	56.8(0.7)	52.3(1.3)	46.2(1.7)
Males	3495(60.7)	662(51.9)	203(54.3)	95(56.2)	290(56.1)	41(27.5)	33(40.1)
Employed Status	1480(16.8)	426(21)	176(41.8)	75(41.6)	103(18.5)	32(20.9)	40(41)
Illiterate	1319(14.8)	495(42.9)	113(29.9)	4(1.87)	279(55.3)	79(46.9)	20(22.2)
No Health Insurance	2477(38.5)	878(66.9)	372(97.1)	40(22.8)	227(45.3)	150(99.4)	89(98.9)
In-patient status at recruitment	2105(34.3)	616(48.6)	119(30.5)	24(13.9)	423(84.5)	39(26.6)	11(12)

**Clinical characteristics**							

NYHA class 1	802(11.9)	105(7.23)	14(3.5)	43(24.9)	27(5.35)	7(4.77)	14(15.1)
NYHA class 2	2548(44.6)	487(37.1)	134(34.5)	67(40)	202(41.5)	28(17.3)	56(59.9)
NYHA class 3	1756(28.80	440(35.3)	152(40)	39(23)	171(33.5)	60(40.2)	18(20.9)
NYHA class 4	714(8.95)	262(20.6)	83(21.7)	20(11.6)	101(19.4)	56(38.4)	2(2.38)
BMI, Kg/m^2^	26.2(0.08)	25.5(0.17)	25.1(0.29)	30.1(0.45)	25.3(0.26)	21.8(0.47)	24.9(0.61)
SBP, mmHg	125(0.3)	124(0.6)	122(1.2)	129(1.8)	119(1.1)	129(2.0)	121(2.5)
DBP, mmHg	75.7(0.18)	78.6(0.38)	78.8(0.79)	82.0(1.19)	75.9(0.70)	84.9(1.27)	74.5(1.64)
Pulse Rate/min	80.3(0.22)	87.6(0.46)	88.0(0.91)	81.2(1.36)	90.4(0.80)	94.6(1.46)	82.3(1.89)
Time of HF diagnosis <1 year	2385(40.5)	715(53.9)	217(56.3)	79(47.3)	263(55.4)	110(71.90)	46(48.3)
Hypertension	3549(64.2)	690(62.1)	252(71.5)	100(60.5)	207(36.9)	104(76.7)	27(39.4)
Diabetes mellitus	1728(28.7)	201(17.2)	41(10.5)	34(18.9)	113(19.8)	12(8.01)	1(1.24)
Dyslipidemia	1812(31.2)	207(21)	59(19.7)	63(36.8)	69(12.2)	11(13.40)	5(6.5)
Chronic Kidney Disease	487(7.12)	45(3.81)	17(4.84)	14(7.98)	11(1.98)	3(2.72)	0(0)
Current Tobacco	554(5.54)	78(3.55)	10(1.83)	29(13.1)	31(4.45)	6(3.99)	2(1.89)
Alcohol	798(7.87)	194(10.6)	61(12.4)	60(30.4)	19(2.36)	25(20)	29(36.9)
Prior Stroke	405(5.88)	55(4.97)	9(2.45)	13(7.73)	28(5.62)	3(2.48)	2(2.69)
Previous MI	1114(17.7)	97(8.26)	4(0.95)	41(23.6)	46(7.93)	6(8.97)	0(0)
HF Hospitalization in Past year	1567(25.2)	420(32.3)	94(24.5)	60(35)	201(39.1)	24(16.7)	41(47.5)
History of COPD	450(6.05)	26(2.22)	5(1.41)	18(10.2)	2(0.34)	1(0.63)	0(0)
MOCA	20.5(0.09)	17.7(0.21)	21.7(0.29)	23.0(0.46)	13.6(0.29)	24.3(1.08)	18.5(0.64)
**Treatments**							

BetaBlockers	3768(66.5)	634(48.4)	112(29.1)	108(63.8)	267(51.9)	105(71.8)	42(49.3)
ACE or ARB	4322(74)	990(77.7)	316(83.3)	127(75.4)	343(67)	124(84.3)	80(90.7)
Aldosterone antagonists	2913(48)	787(59.1)	343(89.4)	81(47.6)	331(66.4)	26(17.8)	6(6.4)
Diuretics	4414(78)	1214(93.7)	360(94)	141(83.8)	489(97.8)	140(93)	84(92.7)
Digoxin	1550(26)	443(31.8)	264(68.5)	32(18.9)	72(14.9)	38(24.3)	37(38.5)
Warfarin	858(14)	222(16.7)	112(29.1)	32(18.6)	66(12.70)	4(2.82)	8(9.32)
**Investigations**							

Creatinine, μmol/L	112(1.15)	129(2.57)	111(5.73)	104(9.98)	139(5.63)	175(14.0)	130(11.4)
Hemoglobin, g/dL	125(0.33)	119(0.77)	119(1.70)	128(2.40)	117(1.27)	129(3.46)	124(2.50)
Sinus Rhythm	3699(75.7)	728(75.2)	239(74.7)	102(81.7)	255(73.6)	60(79)	72(83.1)
Atrial Fibrillation	850(17.3)	154(16.6)	50(15.5)	8(6.23)	74(21.4)	10(10.6)	12(12.4)

Key: SE, standard error; n, number of patients; NYHA, New York Heart Association; BMI, body mass index; SBP, systolic blood pressure; DBP, diastolic blood pressure; HF, heart failure; MI, myocardial infarction; HF, heart failure; COPD, chronic obstructive airway disease; MoCA, Montreal Cognitive Assessment; ACEI, angiotensin converting enzyme inhibitor; ARB, angiotensin II receptor blocker; normal ranges: serum creatinine 30–111 μmol/L; hemoglobin 13.7–16.7 g/dL.

### Demographic characteristics

Details of the demographic and clinical characteristics are in Table 1 (and Supplementary file); showing significant variations between the countries.

Patients from Sudan were older, had the lowest employment status, lived farther away from the hospitals and were recruited more often as in-patients. Majority of the patients from Uganda were females, lived in rural areas and had no health insurance. Patients from Mozambique were the youngest in the African cohort, and nearly all had no health insurance, lived in urban areas and were recruited mostly as out-patients. Majority of the patients from South Africa lived in urban areas and were recruited as out-patients, and nearly all were literate. Nearly all Nigerian patients had no health insurance.

### Clinical characteristics

The data on the use of HF therapies also showed significant variations between the five countries. Beta-blockers use was low in Nigeria (29.1%), which was well-below the African average of 48.4%, and it did not increase during follow-up (28.1%). The use of angiotensin converting enzyme inhibitors or angiotensin II receptor blockers in the African countries was in general good and above the overall INTER-CHF average, but the dosages used were lower than the global average, and the on-target dosages were poorly achieved by the patients from Sudan and Uganda. History of prior percutaneous coronary interventions was present in less than 1% of patients from Nigeria who also had the lowest history of prior myocardial infarction, while only 1.2% of the South African patients received an Implantable Cardioverter Defibrillator and none of the patients received Cardiac Resynchronised Therapy.

### Etiology of heart failure

Majority of patients (53.7%) had HFrEF, followed by those with HFmrEF (30.1%) and HFpEF (16.2%). Patients from Nigeria had the highest prevalence of HFrEF (79.5%), those from Sudan had the highest for HFmrEF (27.5%), and HFpEF was highest in Uganda (31.0%). The details on the etiologies of HF in the five African countries are presented in Table 2 and Figure 1b. The most common etiology of HF in the African cohort as well as in Nigeria, Uganda and Mozambique was hypertensive heart disease (HHD), followed by ischemic heart disease (IHD), which was dominant in South Africa and Sudan. Rheumatic heart disease was an important cause of HF in Sudan, Uganda and Mozambique but rare in Nigeria and South Africa.

**Table 2:** Etiologies of Heart Failure.

Variables	Africa Overall N = 1294	Nigeria N = 383	South Africa N = 169	Sudan N = 501	Uganda N = 151	Mozambique N = 90

Hypertensive HD	35	65.1	13.6	11.7	46.9	24.7
Ischemic HD	20	3.01	44.9	25.3	1.09	1.27
Idiopathic – DCM	14.1	10.6	8.61	20.9	22.2	18.3
Rheumatic HD	7.2	5.3	3.3	16.8	13	19.3
Valvular Non-Rheumatic HD	2.3	2.0	2.2	1.3	7.8	2.4
HIV Cardiomyopathy	0.7	0.2	3.3	0	0.83	3.8
Alcohol/Drug induced CMP	0.66	0.68	1.1	0.43	0	2.4
EMF	0.26	0	0.45	0	1.8	2.2
PPCM	0.14	0.58	0.60	0.17	0	0.38
Post Chemotherapy HD	0.12	0	0.69	0	0	0
Tuberculosis related HD	0.09	0	0.51	0	0	0.82
Congenital HD	0.08	0.26	0	0.17	0.58	0
Endocrine/Metabolic	5.4	0.73	3.5	11	1.9	0.9
Other causes	13.3	11.6	16.2	11.4	3.9	23.5
Unknown etiology	0.6	0	1.1	0.87	0	0

Key: HD, heart disease; DCM, dilated cardiomyopathy; HIV, human immunodeficiency virus; CMP, cardiomyopathy; EMF, endomyocardial fibrosis; PPCM, peripartum cardiomyopathy. Values are expressed as proportions in percentages.

The mean MoCA score was highest in Uganda (24.3 ± 1.1) and lowest in Sudan (13.6 ± 0.3), while the African average was 17.7 ± 0.2.

### Outcomes

**Figure 2:**
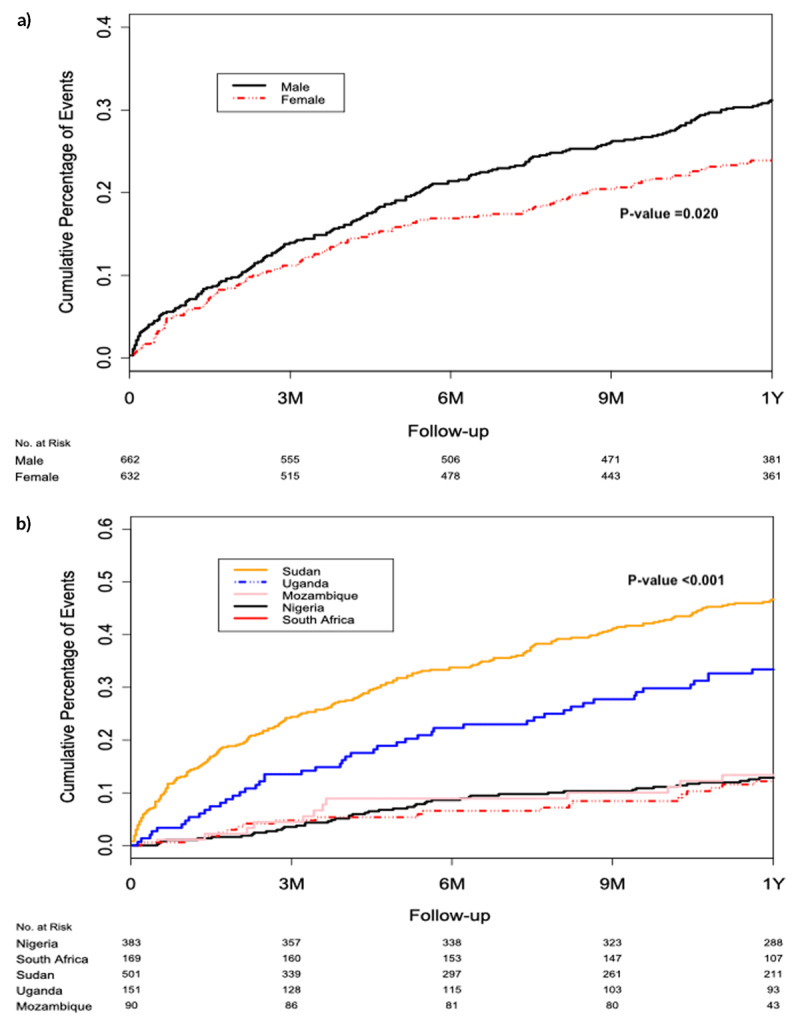
Kaplan Meir curves: **(2a)** Death outcome by Region. **(2b)** Death outcome by Sex. Legend: Kaplan Meier survival cures describing mortality pattern in the five African countries (Figure 2a) and in males and females (Figure 2b), over one year.

Details of outcomes are presented in Tables 3 and 4 and Figure 2a and 2b. The one-year all-cause mortality rate in the five countries was 26.4%, which was highest in Sudan (42.7%) and lowest in South Africa (11.7%). Further analysis showed that death rates were higher in males than females (aHR: 1.42 [95% CI 1.06–1.92]; p = 0.02) (Figure 2b).

**Table 3:** Outcomes.

Variables	Overall Africa N = 1294	Nigeria N = 383	South Africa N = 169	Sudan N = 501	Uganda N = 151	Mozambique N = 90	P-VALUE

**Death at Year 1**	26.4	12.3	11.8	42.7	32.5	13.3	<0.0001*
**Hospitalization at Year 1**	24.3	20.6	16.6	37.3	6.0	13.3	<0.0001*
**Death or Hospitalization at Year 1**	39	25.3	21.9	59.7	35.1	21.1	<0.0001*
**Cardiac Death at Year 1**	9.0	7.3	7.1	13.6	0.7	7.8	<0.0001*
**Non-Cardiac Death at Year 1**	2.9	1.6	3.6	4.4	0	3.3	0.0038*
**Unknown Death at Year 1**	14.6	3.4	1.2	24.8	31.8	2.2	<0.0001*

Key: Values are expressed as proportions in percentages; * p-value statistically significant.

**Table 4:** Correlates of mortality.

Variables	Unadjusted HR (95% CI)	P-value	Adjusted HR (95% CI)	P-Value

**Model 1**				

Age (Unit = 10), years	1.10 (1.03–1.17)	0.007*	1.10 (1.01–1.21)	0.037*
Sex: Male vs Female	1.35 (1.09–1.68)	0.006*	1.42 (1.06–1.92)	0.020*
**Model 2**				

Age (Unit = 10), years	1.10 (1.03–1.17)	0.007*	1.09 (0.98–1.20)	0.102
Sex: Male vs Female	1.35 (1.09–1.68)	0.006*	1.324 (0.96–1.82)	0.084
SBP (Unit = 10), mmHg	0.86 (0.82–0.91)	<0.001*	0.86 (0.81–0.93)	<0.001*
Chronic Kidney Disease: Yes vs No	1.62 (1.01–2.61)	0.047	2.790 (1.55–5.02)	<0.001*
History of Diabetes Mellitus: Yes vs No	1.45 (1.11–1.89)	0.007	1.12 (0.77–1.64)	0.552
NYHA Class:(3 or 4 vs 1 or 2)	1.71 (1.37–2.14)	<0.001*	1.04 (0.76–1.43)	0.788
In patient vs Out Patient	3.66 (2.89–4.64)	<0.001*	2.92 (2.09–4.10)	<0.001*
Prior hospitalization for HF: Yes vs No	1.74 (1.41–2.16)	<0.001*	1.42 (1.07–1.89)	0.016*
Ischemic Etiology: Yes vs No	1.27 (0.98–1.64)	0.075	0.96 (0.67–1.38)	0.813
Depressed LV function: Yes vs No	1.08 (0.83–1.41)	0.580	0.91 (0.67–1.24)	0.552
Valve Disease: Yes vs No	2.54 (1.88–3.44)	<0.001*	1.88 (1.33–2.66)	<0.001*
COPD: Yes vs No	0.63 (0.26–1.52)	0.303	0.82 (0.30–2.26)	0.700
BMI Kg/m^2^	0.96 (0.94–0.98)	<0.001*	1.00 (0.97–1.02)	0.832
Smoking: Current vs former or Never	0.75 (0.46–1.23)	0.256	0.78 (0.40–1.50)	0.451
On Beta Blocker: Yes vs No	1.01 (0.81–1.25)	0.950	0.80 (0.59–1.08)	0.152
On ACEI/ARB: Yes vs No	0.68 (0.54–0.85)	0.001	0.86 (0.62–1.19)	0.355
On Aldosterone Inhibitor: Yes vs No	0.92 (0.74–1.14)	0.459	0.82 (0.60–1.12)	0.222
On Digoxin: Yes vs No	0.61 (0.48–0.77)	<0.001*	0.69 (0.49–0.97)	0.034*

Key: HR, hazard ratio; NYHA, New York Heart Association; BMI, body mass index; SBP, systolic blood pressure; COPD, chronic obstructive airway disease; ACEI, angiotensin converting enzyme inhibitor; ARB, angiotensin II receptor blocker; * p-value statistically significant.

HF hospitalization was highest in Sudan (37.3%) and lowest in Uganda (6.0%), and the composite outcome of death or HF hospitalization was also highest in Sudan (59.7%) and lowest in Mozambique (21.1%). The commonest recorded cause of death was cardiac, but the specific causes could not be ascertained for the majority of deaths.

Correlates of mortality were determined using Cox proportional regression models and presented in Table 4. In the model comprising age and sex, every 10-year unit increase in age increased the risk of mortality by 10.2% (p = 0.037) while male sex was associated with 42% higher risk of mortality than females (p = 0.020). In the second model comprising eighteen variables, four of them (chronic kidney disease, in-patient status, prior HF hospitalization and valve disease etiology) were independently associated with higher risk of mortality while a 10 mmHg unit increase in systolic BP and digoxin use were independently associated with lower mortality risk.

## Discussion

In the present study, we described the clinical profiles and outcomes of 1294 chronic and acutely decompensated HF patients recruited from Nigeria, Sudan, Mozambique, Uganda and South Africa in the INTER-CHF study. The main outcomes assessed were all-cause deaths, HF hospitalizations and the composite of the deaths or HF hospitalizations over one year. We classified HF into HFrEF, HFmrEF and HFpEF, in accordance with the recommendation of the most recent ESC guidelines, as there is paucity of such data in Africa [8]. We found significant differences between the patients in terms of patients’ demographic and clinical characteristics, HF etiology, treatment patterns and outcomes stratified by countries. The details of comparisons between the African and other countries in the INTER-CHF cohort have already been published [3,4,5]. Therefore, in this paper we focus on data of the five African countries, each representing an African region.

**Figure 3:**
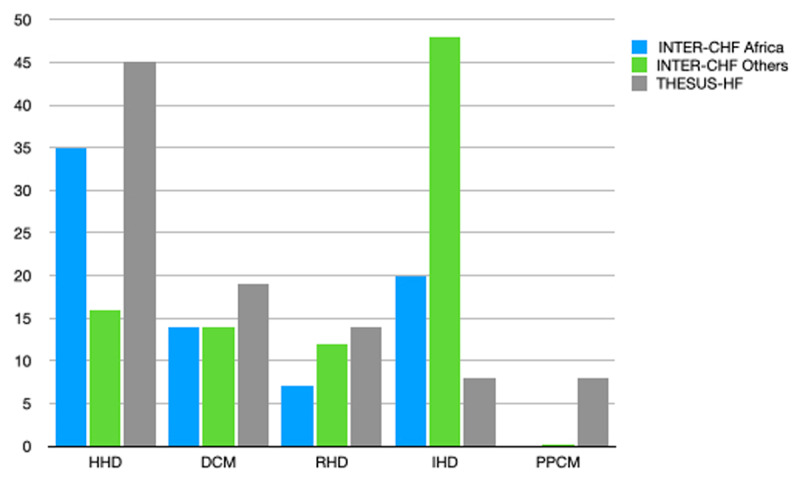
Etiologies of HF in INTER-CHF Africa and THESUS-HF cohorts. Legend: Common etiologies of HF in the INTER-CHF (Africa and other regions) and THESUS-HF cohorts. HHD, hypertensive heart disease; DCM, dilated cardiomyopathy; RHD, rheumatic heart disease; IHD, ischemic heart disease; PPCM, peripartum cardiomyopathy.

The commonest etiologies of HF were HHD (35%) followed by IHD (20%). The diagnosis of IHD was mostly based on clinical, troponin blood levels and echocardiographic and electrocardiographic findings, and only rarely on invasive investigations, given that only 6% had percutaneous coronary interventions. Although it is conceivable that some cases of IHD in the cohort were misdiagnosed as HHD, it is important to appreciate that the LV geometric phenotype of HHD with eccentric LV hypertrophy and systolic dysfunction is common among Africans as previously reported among Nigerians [9]. Our results differ significantly from what was found in the other INTER-CHF study regions (total number of patients = 4,529) and THESUS–HF study (total number of patients = 1,006) (Figure 3) [3,4,5]. HHD, dilated and peripartum cardiomyopathies and rheumatic heart disease were all more frequent in the THESUS-HF cohort, while IHD was more frequent in the INTER-CHF cohort. These differences could perhaps be explained by the fact that THESUS-HF recruited acutely decompensated HF patients at an earlier time period (2007-2010) in the African epidemiological transition than the INTER-CHF (2012-2014), and one-fifth of the participants was recruited from Kano (Nigeria) which has the highest reported incidence of peripartum cardiomyopathy in the world [3,4,5,10].

The MoCA is an objective test of cognitive function in HF patients, and is associated with poor outcomes [11]. While the exact mechanisms for decline in cognitive function in HF remain unclear, several possibilities include chronic or intermittent cerebral hypoperfusion and/or microemboli from possible LV thrombi formation [12]. Our results showed that the MoCA score for the African cohort (17.7) was below the overall average (20.5), and was highest in Uganda (24.3) and lowest in Sudan (13.6), which are all below the normal cut-off value of 26. The implication of the low MoCA score is huge if interpreted in the context of the prevailing young African HF population, in their productive years. However, the MoCA should be cautiously interpreted in Africa because of the relatively low literacy level. This is because the relatively low score might be a reflection of the low literacy level, rather than true impairment of cognitive function, since educational level can influence the domains assessed in MoCA test and the overall score, particularly given the fact that the test had to occasionally be translated to local languages. For these reasons, we propose that in Africa, the MoCA should be selectively applied to only literate HF patients, given its dependency on educational level. To the best of our knowledge, the INTER-CHF is the first study to describe the cognitive function in HF patients in Africa.

In the present study, Sudan had the highest incidence of all-cause (43%) deaths, HF hospitalization (37%) and the composite of deaths or HF hospitalization (60%) at one year, than all the other countries. It was followed by Uganda, while the other three countries had comparatively similar outcomes. The Kaplan-Meier curves illustrated that the mortality rates in Sudan and Uganda increased earlier during follow-up and persisted without a plateau, whereas deaths in other countries seemed to peak around the sixth follow-up month. The worse clinical outcomes in Sudan and Uganda are most likely due to the significantly higher frequencies of in-patient recruitment status in Sudan (84.5%), and of NYHA functional classes III or IV (78.6%) at recruitment and valve disease etiology (7.8%) in Uganda, which are all independent correlates of mortality in the present study. In partial agreement with our findings, the REPORT-HF (International Registry to Assess Medical Practice with Longitudinal Observation for Treatment of Heart Failure) registry reported old age, chronic kidney disease and presence of valvular heart disease, amongst other covariates, as the predictors of mortality in HF patients [13]. Investigators of the ESC Long-term HF Registry also reported that older age, NYHA class and chronic kidney disease were independently associated with a worse outcome regardless of LVEF category [14]. In addition, non-cardiovascular co-morbidities, such as depression, peripheral artery disease and hepatic dysfunction, were predictive of mortality at one year in the different LVEF categories [14]. In the larger INTER-CHF study, several clinical and demographic variables were independently associated with death within one year, including the ones observed in this post-hoc analysis of the African data [4]. These included age (aHR 1·1; 95% CI 1·05–1·17), systolic BP (aHR 0·92 per 10 mm Hg increase; 0·88–0·96), history of chronic kidney disease (aHR 1·9; 1·5–2·5), NYHA functional classes III or IV (aHR 1·4; 1·2–1·7), enrolment as hospital in-patient (aHR 1·9; 1·6–2·2), admission for HF in the previous year (aHR 1·6; 1·3–1·9) and valve disease etiology (aHR 1·6; 1·3–1·9) [4].

Of all the pharmacologic treatments for HF that the patients received, only digoxin was associated with a lower risk for mortality, with an aHR of 0.69. Most patients in Nigeria (69%) received digoxin, which was considerably higher than the average for Sudan (15%), Africa (32%) and the overall INTER-CHF cohort (26%). Prescriptions for beta-blockers in the study was poor while those for angiotensin-converting enzyme inhibitors, angiotensin II receptor blockers and mineralocorticoid receptor antagonists were fair at baseline. However, only 4%, 6%, 13% and 8% of patients received beta-blockers, angiotensin-converting enzyme inhibitors, angiotensin II receptor blockers and mineralocorticoid receptor antagonists at the recommended target doses in the study, which probably explains why these agents failed to improve survival in the African cohort. Medications associated with mortality at one year in the larger INTER-CHF study were angiotensin-converting enzyme inhibitors or angiotensin receptor blockers (aHR 0·8; 0·7–0·9), and digoxin use at enrolment (aHR 0·8; 0·7–0·9) [4]. The poor treatment of HF patients in Africa is obviously related to the poor healthcare financing and low medical insurance coverage, as previously discussed in the main INTER-CHF papers [2,3,4]. An important strategy to improve the poor HF outcomes in Africa would be to strengthen the healthcare systems and improve healthcare access through universal insurance coverage, as illustrated by the data from South Africa.

In ambulatory patients with chronic HFrEF that received angiotensin-converting enzyme inhibitors, but not optimal doses of beta-blockers or mineralocorticoid receptor antagonists, discontinuation of digoxin therapy increased the risk of adverse outcomes [15,16,17]. In the OPTIMIZE-HF (Organized Program to Initiate life-saving Treatment in Hospitalized Patients with Heart Failure) registry, discontinuation of pre-admission digoxin therapy among hospitalized older patients with HFrEF on more contemporary guideline-directed medical therapies, was associated with significantly higher risks of HF readmission (HR 1.21), all-cause readmission (HR 1.16), and the combined endpoint of HF readmission or all-cause mortality (HR 1.20) [17]. However, a randomized prospective trial would be needed to confirm the impact of digoxin on clinical outcomes in HFrEF patients receiving contemporary HF treatments.

Our results showed that male sex increased the risk of death, when adjusted for age (aHR 1.4). However, this effect of male sex disappeared when it was adjusted for several other variables. In the ESC HF Long-Term Registry, rates of all-cause mortality were significantly lower in women than in men (7.1% vs. 8.7%) with HFrEF, as were rates of all-cause hospitalization (21.9% vs. 27.3%) and there were no differences in causes of death. However, sex was not an independent predictor of one-year all-cause mortality, in agreement with our finding [14].

### Limitations

The strengths and weaknesses of the INTER-CHF study have already been previously published [3]. Important was the use of a standardized protocol which ensured a common approach to recruitment and documentation of patient characteristics, that allowed direct comparisons of data among the regions and countries. However, we enrolled patients with a clinical HF diagnosis and who satisfied the Boston criteria for HF, because we wanted to enroll typical real-world HF patients HF in the regions with less developed healthcare systems. In most African centers and some in other regions, natriuretic peptides were not routinely measured to diagnose or follow-up HF patients. Thus, it is possible that a few patients who had LVEF above 40% and who did not have documented high B-type natriuretic peptide, did not in fact have HFpEF. Secondly, the simplistic design of the INTER-CHF study in terms of determination of HF etiology is an important limitation. The reliance on physician discretion in relation to determination of etiology, without clear case definitions in the protocol, and the existing regional infrastructure variation, could allow for selection bias. However, the findings of this study are still very relevant and hypothesis-generating. Another limitation was the high frequency of ‘unknown’ causes of death in up to one-quarter of the deceased cases in Sudan and one-third of those in Uganda, likely because of prohibiting cultural reasons against post-mortem examinations or even consenting to verbal autopsy, as previously reported from Africa [18]. Finally, we acknowledge that our data was dominated by recruitment from Sudan and Nigeria, which should be considered in the interpretation of the results.

## Conclusions

This is the largest HF study thus far in Africa that included acutely decompensated and chronic HF patients from the West, East, North, Central and South African subregions. The clinical profiles and outcomes of 1,294 HF patients recruited from Nigeria, Sudan, Mozambique, Uganda and South Africa were described. The commonest etiologies of HF were HHD and IHD. The patterns of HF treatment and co-morbidities varied widely between the countries. Prescriptions for HF therapies were poor particularly for beta-blockers and the device therapies were largely unavailable. Sudan had the highest incidence of all-cause and cardiac deaths, as well as HF hospitalization and the composite of deaths or HF hospitalization at one year, than all the other countries, most likely due to their high in-hospital recruitment pattern. Of all the pharmacologic treatments for HF that the patients received, only digoxin significantly lowered the risk for mortality. MoCA score for the African cohort was below the overall average for INTER-CHF study due to the low literacy level, and was highest in Uganda and lowest in Sudan. To the best of our knowledge, the INTER-CHF is the first study to describe the cognitive function in HF patients in Africa. However, the lack of clear case definitions in relation to HF etiology in the study protocol is a noteworthy limitation.

## Data Accessibility Statement

Data used in the present study has not been made publicly available, but on specific request to the corresponding author (KK).

## Additional File

The additional file for this article can be found as follows:

10.5334/gh.940.s1 Supplement data. Clinical Profiles and Outcomes of Heart Failure in Five African Countries: Results from INTER-CHF Study.
