# Supplementary material for: Clinical Profiles and Outcomes of Heart Failure in Five African Countries: Results from INTER-CHF Study
Source: Glob Heart. 2021 Jul 30;16(1):50. doi: 10.5334/gh.940 (PMC8323533; doi:10.5334/gh.940)
Supplement: Supplement data. — Clinical Profiles and Outcomes of Heart Failure in Five African Countries: Results from INTER-CHF Study. [file gh-16-1-940-s1.pdf]

<u>Supplementary data</u>

Title: Clinical Profiles and Outcomes of Heart Failure in Five African Countries: Results from INTER-CHF Study

<u>Figure S1: Healthcare indices of 5 African countries</u>

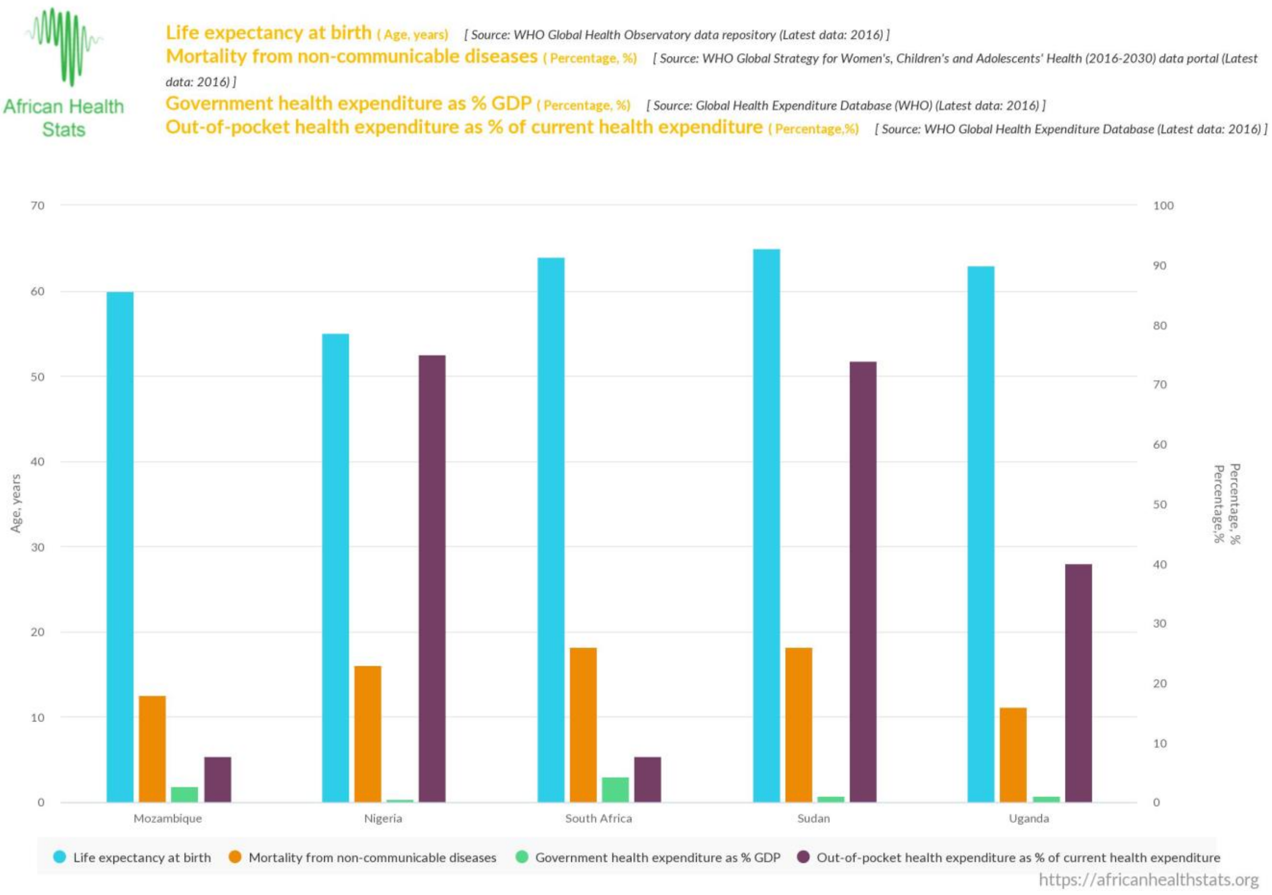

Table S1: Government healthcare funding and literacy rate in 5 African countries

Country	Government-funded health system/insurance[b]	Literacy rate[a]
**Nigeria**	Non-universal	62.0%
**Sudan**	Non-universal	60.7%
**Mozambique**	Non-universal	60.7%
**Uganda**	Non-universal	76.5%
**South Africa**	Universal	87.0%

Key: [a], accessed at https://worldpopulationreview.com/country-rankings/literacy-rate-by-country (on 10 April 2021); [b], accessed at https://en.wikipedia.org/wiki/Health_care_systems_by_country (on 10 April 2021) .

Table S2: Baseline characteristics of patients in the 5 African countries

Variables	Overall Mean(SE)or(%) N=5823	Africa Overall Mean(SE)or(%) N=1294	Nigeria Mean(SE)or(%) N=383	SouthAfrica Mean(SE)or(%) N=169	Sudan Mean(SE)or(%) N=501	Uganda Mean(SE)or(%) N=151	Mozambique Mean(SE)or(%) N=90	P-Value for trend
**Demographic characteristics**								
Age, years	59.3(0.2)	53.4(0.4)	50.8(0.8)	53.3(1.2)	56.8(0.7)	52.3(1.3)	46.2(1.7)	<0.001
Males vs Females	60.7	51.9	54.3	56.2	56.1	27.5	40.1	<0.001
Employed Status	16.8	21	41.8	41.6	18.5	20.9	41	<0.001
Illiterate	14.8	42.9	29.9	1.87	55.3	46.9	22.2	<0.001
Rural residence	35.5	31.9	25.3	10.2	36.2	68.6	11.8	<0.001
No Health Insurance	38.5	66.9	97.1	22.8	45.3	99.4	98.9	<0.001
In patient status at recruitment	34.3	48.6	30.5	13.9	84.5	26.6	12	<0.001
**Clinical characteristics**								
Mean NYHA class	2.39(0.01)	2.69(0.02)	2.80(0.04)	2.21(0.06)	2.67(0.04)	3.11(0.07)	2.13(0.09)	<0.001
NYHA class 1	11.9	7.23	3.5	24.9	5.35	4.77	15.1	<0.001
NYHA class 2	44.6	37.1	34.5	40	41.5	17.3	59.9	<0.001
NYHA class 3	28.8	35.3	40	23	33.5	40.2	20.9	<0.001
NYHA class 4	8.95	20.6	21.7	11.6	19.4	38.4	2.38	<0.001
BMI, Kg/m^2	26.2(0.08)	25.5(0.17)	25.1(0.29)	30.1(0.45)	25.3(0.26)	21.8(0.47)	24.9(0.61)	<0.001
SBP, mmHg	125(0.3)	124(0.6)	122(1.2)	129(1.8)	119(1.1)	129(2.0)	121(2.5)	<0.001

Variables	Overall Mean(SE)or(%) N=5823	Africa Overall Mean(SE)or(%) N=1294	Nigeria Mean(SE)or(%) N=383	SouthAfrica Mean(SE)or(%) N=169	Sudan Mean(SE)or(%) N=501	Uganda Mean(SE)or(%) N=151	Mozambique Mean(SE)or(%) N=90	P-Value for trend
DBP, mmHg	75.7(0.18)	78.6(0.38)	78.8(0.79)	82.0(1.19)	75.9(0.70)	84.9(1.27)	74.5(1.64)	<0.001
Pulse Rate/min	80.3(0.22)	87.6(0.46)	88.0(0.91)	81.2(1.36)	90.4(0.80)	94.6(1.46)	82.3(1.89)	<0.001
Time of HF diagnosis <1 year	40.5	53.9	56.3	47.3	55.4	71.9	48.3	<0.001
Hypertension	64.2	62.1	71.5	60.5	36.9	76.7	39.4	<0.001
Diabetes mellitus	18.7	17.2	10.5	18.9	19.8	8.01	1.24	<0.001
Dyslipidemia	31.2	21	19.7	36.8	12.2	13.4	6.5	<0.001
Chronic Kidney Disease	7.12	3.81	4.84	7.98	1.98	2.72	0	<0.001
Tobacco Use (ever)	5.54	3.55	9.13	38.8	13.7	14.8	7.1	<0.001
Alcohol Use (any)	7.87	10.6	12.4	30.4	2.36	20	36.9	<0.001
Prior Stroke	5.88	4.97	2.45	7.73	5.62	2.48	2.69	0.030
History of MI	17.7	8.26	0.95	23.6	7.93	8.97	0	<0.001
CHF Hospitalization in Past year	25.2	32.3	24.5	35	39.1	16.7	47.5	<0.001
History of COPD	6.05	2.22	1.41	10.2	0.34	0.63	0	<0.001
MOCA	20.5(0.09)	17.7(0.21)	21.7(0.29)	23.0(0.46)	13.6(0.29)	24.3(1.08)	18.5(0.64)	<0.001
**Treatments**								
Baseline BetaBlocker	66.5	48.4	29.1	63.8	51.9	71.8	49.3	<0.001

Variables	Overall Mean(SE)or(%) N=5823	Africa Overall Mean(SE)or(%) N=1294	Nigeria Mean(SE)or(%) N=383	SouthAfrica Mean(SE)or(%) N=169	Sudan Mean(SE)or(%) N=501	Uganda Mean(SE)or(%) N=151	Mozambique Mean(SE)or(%) N=90	P-Value for trend
Followup BetaBlocker	66.7	51.3	28.1	66.2	56.5	74.9	63.1	<0.001
Beta Blocker-On target dose	6.39	4.03	0.88	21	0.4	1.49	1.86	<0.001
Baseline ACE/ARB	74	77.7	83.3	75.4	67	84.3	90.7	<0.001
Followup ACE/ARB	72.2	76.8	84.3	78.1	63.7	83	89.7	<0.001
Baseline ACE Inhibitor	48.4	58.6	53.1	60.5	54.7	76	87.9	<0.001
ACE Inhibitor-On target dose	7.97	5.71	3.75	12.4	0	0	30.4	<0.001
Angiotensin Receptor Blocker	24.1	18.8	30	14.4	12.9	7.82	1.25	<0.001
Angiotensin Receptor Blocker-On target dose	17.4	13.2	13.2	37.3	0	18.3	100	<0.001
Baseline Aldosterone Inhibitors	48	59.1	89.4	47.6	66.4	17.8	6.4	<0.001
Followup Aldosterone Inhibitors	46.5	57.4	85.7	50.3	62.7	18.9	7.57	<0.001
Aldosterone Inhibitors-On target dose	5.94	7.78	7.49	21.4	4.02	15.2	39.7	<0.001

Variables	Overall Mean(SE)or(%) N=5823	Africa Overall Mean(SE)or(%) N=1294	Nigeria Mean(SE)or(%) N=383	SouthAfrica Mean(SE)or(%) N=169	Sudan Mean(SE)or(%) N=501	Uganda Mean(SE)or(%) N=151	Mozambique Mean(SE)or(%) N=90	P-Value for trend
Diuretic	78	93.7	94	83.8	97.8	93	92.7	<0.001
Baseline Digoxin	26	31.8	68.5	18.9	14.9	24.3	38.5	<0.001
Baseline Long acting Nitrate	15.1	4.71	1.22	5.29	6.4	0.66	3.82	<0.001
Baseline Warfarin	14	16.7	29.1	18.6	12.7	2.82	9.32	<0.001
Baseline Aspirin	56.2	45.9	51.8	41.8	48.9	23.5	4.88	<0.001
Prior coronary angioplasty or stent	12.4	6.23	0.43	15.1	7.16	0.7	0	<0.001
Cardiac Rhythm Device	4.09	1.82	0.33	5.33	0.69	0.54	0	<0.001
--Pacemaker	1.66	1.49	0.32	3.23	0.68	0.5	0	0.004
--ICD	0.02	0.14	0	1.16	0	0	0	
--CRT	0.41	0.07	0	0	0	0	0	
--CRT and ICD	0.01	0.14	0	0	0	0	0	
Cardiac Surgery	7.63	3.63	0.52	16.1	1.63	0.67	9.81	<0.001
**Investigations**								
Sodium	138(0.07)	137(0.16)	138(0.30)	138(0.51)	134(0.31)	138(0.73)	138(0.59)	<0.001
Creatinine	112(1.15)	129(2.57)	111(5.73)	104(9.98)	139(5.63)	175(14.0)	130(11.4)	<0.001
Hemoglobin	125(0.33)	119(0.77)	119(1.70)	128(2.40)	117(1.27)	129(3.46)	124(2.50)	<0.001
ECG - Sinus Rhythm	75.7	75.2	74.7	81.7	73.6	79	83.1	0.161

Variables	Overall Mean(SE)or(%) N=5823	Africa Overall Mean(SE)or(%) N=1294	Nigeria Mean(SE)or(%) N=383	SouthAfrica Mean(SE)or(%) N=169	Sudan Mean(SE)or(%) N=501	Uganda Mean(SE)or(%) N=151	Mozambique Mean(SE)or(%) N=90	P-Value for trend
ECG - Atrial Fibrillation	17.3	16.6	15.5	6.23	21.4	10.6	12.4	<0.001
ECG - Atrial Flutter	0.77	1.27	1.18	0.72	1.2	1.47	1.27	0.987
HF with reduced LVEF (<40%)	49.8	53.7	79.5	76.1	62.1	59.6	74.6	<0.001
HF with mid-range LVEF (40-49%)	30.2	30.1	2.1	6.1	27.5	9.4	19.6	<0.001
HF with preserved LVEF(>= 50%)	20	16.2	18.4	17.8	10.4	31	5.8	<0.001
